# The role of hypoxia in inflammatory disease (Review)

**DOI:** 10.3892/ijmm.2015.2079

**Published:** 2015-01-17

**Authors:** JOHN BIDDLESTONE, DANIEL BANDARRA, SONIA ROCHA

**Affiliations:** 1 Centre for Gene Regulation and Expression, College of Life Sciences, University of Dundee, Dundee DD1 5EH, UK; 2 Plastic Surgery Training Programme, NHS Scotland, Scotland, UK

**Keywords:** hypoxia, inflammation, inflammatory bowel disease, rheumatoid arthritis, colorectal cancer

## Abstract

Mammals have developed evolutionarily conserved programs of transcriptional response to hypoxia and inflammation. These stimuli commonly occur together *in vivo* and there is significant crosstalk between the transcription factors that are classically understood to respond to either hypoxia or inflammation. This crosstalk can be used to modulate the overall response to environmental stress. Several common disease processes are characterised by aberrant transcriptional programs in response to environmental stress. In this review, we discuss the current understanding of the role of the hypoxia-responsive (hypoxia-inducible factor) and inflammatory (nuclear factor-κB) transcription factor families and their crosstalk in rheumatoid arthritis, inflammatory bowel disease and colorectal cancer, with relevance for future therapies for the management of these conditions.

## 1. Introduction

Oxygen (O_2_) constitutes 20.8% of the atmospheric air, and is the third-most abundant element in the universe, after hydrogen and helium. It is not only a key component of all major biomolecules of living organisms, but also a key constituent of inorganic compounds. Oxygen homeostasis is crucially important to maintain the survival of all vertebrate species (1). Therefore, organisms developed a way to coordinate the oxygen levels in the intracellular compartments in order to maintain homeostasis. When these mechanisms fail, and the intracellular concentration of oxygen decreases, a stress condition called hypoxia is created. Hypoxia can be defined as a condition lacking the necessary oxygen to meet metabolic requirements. The level at which this is reached will vary depending on the metabolic requirements of the cell. Hypoxia is a relevant physiological stress associated with many processes, such as adaptation to high altitudes or human diseases (e.g, cancer) (2). The hypoxia-inducible factors (HIFs) are a family of transcription factors whose levels are regulated in response to hypoxic stimuli, and when active can enact a transcriptional program that allows the cell to respond to the hypoxic environment.

Another important physiological stress is inflammation. Inflammation represents a protective attempt to eliminate pathogens and initiate the healing process of a wound. As in hypoxia, cells have evolved sophisticated mechanisms to control the inflammatory response to pathogens. A key element of these mechanisms is a family of transcription factors termed nuclear factor κ-light-chain-enhancer of activated B cells (NF-κB). NF-κB is composed of several family members that activate signalling pathways in response to a variety of stimuli (such as virus, bacteria or cytokines) which ultimately engage a complex transcriptional program, allowing the cell to respond to this environmental stress (3).

Several diseases, including rheumatoid arthritis (RA), inflammatory bowel disease and colorectal cancer (CRC) result from the deregulation of the hypoxia and inflammation pathways (4–6). Consequently, recent scientific research has been focussed on attempting to understand how these pathways are regulated, crosstalk and respond in disease. In this review, we describe the current understanding of the role of the HIF and NF-κB transcription factor families in response to hypoxia and inflammation and discuss their crosstalk in RA, inflammatory bowel disease and CRC, with relevance for future therapies for the management of these conditions.

## 2. The HIF system

**Figure 1:**
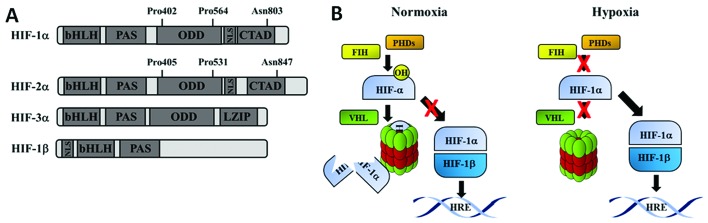
(A) Schematic diagram of HIF proteins. Boxes represent different protein domains. The hydroxylation sites for HIF-1α and HIF-2α are noted above the schematic structure. (B) Schematic diagram of HIF pathway. In the presence of oxygen (normoxia), c bind to HIF-1α and catalyse the hydroxylation of proline residues. Once hydroxylated, HIF-1α binds rapidly to the vHL, which results in its polyubiquitination. This targets HIF-1α for proteasome-mediated degradation. In the presence of low oxygen (hypoxia), HIF-1α is stabilized and can translocate to the nucleus. HIF-1α dimerises with its partner HIF-1β and transactivates target genes containing hypoxia response elements (HREs). HIF, hypoxia-inducible factor; vHL, von Hippel Lindau; bHLH, basic helix-loop-helix; CTAD, C-terminal transactivation domain; LZIP, leucine zipper; NLS, nuclear localisation signal; ODD, oxygen-dependent domain; PAS, Per/ARNT/Sim domain.

The HIFs are a family of transcription factors that sense changes in environmental oxygen and orchestrate a transcriptional program, which forms an important part of the cellular response to the hypoxic environment. HIF-1 was first identified over 20 years ago through studies of erythropoietin gene expression (7). HIF is a heterodimeric transcription factor that consists of a constitutively expressed HIF-1β subunit and an O_2_-regulated HIF-α subunit (8). Three isoforms of HIF-α have been identified since these initial studies (HIF-1α, -2α and -3α) (Fig. 1A). The HIF-α isoforms are all characterized by the presence of basic helix-loop-helix (bHLH)-Per/ARNT/Sim (PAS) and oxygen-dependent degradation (ODD) domains (Fig. 1A). Both HIF-1α and HIF-2α have important cellular functions as transcription factors with some redundancy in their targets (9,10). HIF-2α protein shares sequence similarity and functional overlap with HIF-1α, but its distribution is restricted to certain cell types, and in some cases, it mediates distinct biological functions (11). HIF-3α is the most recently discovered isoform. The regulation of HIF-3α expression is complex in comparison to HIF-1α and HIF-2α with several splice variants that can function as a competitive inhibitors of the HIF-α-HIF-1β interaction (12,13), or by directly activating target genes in hypoxia that mediate both hypoxia dependent and independent functions. The role of HIF-3α in the cellular response to hypoxia remains an active area of study (14). Several splice variants of HIF-1β [also known as aryl hydrocarbon receptor nuclear translocator (ARNT)] have been identified (15,16). Though their exact functions are not known, at least one splice variant has been associated with poor prognosis in oestrogen receptor-negative breast cancer (17).

## 3. HIF regulation by οxygen

The regulation of the HIF-α subunits by oxygen occurs mainly at the post-transcriptional level, and is mediated by hydroxylation-dependent proteasomal degradation (Fig. 1B). In well-oxygenated cells, HIF-α is hydroxylated in its ODD. For HIF-1α this is at prolines (Pro)402 and Pro564 (18), whereas HIF-2α is hydroxylated at Pro405 and Pro531 (Fig. 1B) (19). Proline hydroxylation is catalysed by a class of dioxygenase enzymes called prolyl hydroxylases (PHDs). There are three known PHDs, 1–3, all of which have been shown to hydroxylate HIF-1α. PHD2 has a higher affinity for HIF1α, whereas PHD1 and PHD3 have higher affinity for HIF-2α (20,21). All PHDs require Fe^2+^ and α-ketoglutarate (α-KG) as co-factors for their catalytic activity and have an absolute requirement for molecular oxygen as a co-substrate, making their activity reduced in hypoxia (22–25).

Prolyl-hydroxylation of HIF-α attracts the von Hippel-Lindau (vHL) tumour suppressor protein, which recruits the Elongin C-Elongin B-Cullin 2-E3-ubiquitin-ligase complex, leading to the Lys48-linked poly-ubiquitination and proteasomal degradation of HIF-1α (Fig. 1B) (26–28). Interestingly, PHDs have also been shown to be able to sense amino acid availability through α-KG oscillations (29), and the centrosomal protein Cep192 has been described as a hydroxylation target for PHD1 (30), indicating an additional function for these enzymes as nutrient sensors and regulators of cell cycle progression. Both PKM2 and HCLK2 have also both recently been described as new hydroxylation targets for PHD3 (31,32).

In hypoxia the PHDs are inactive, or have reduced activity, since they require molecular oxygen as a cofactor. Under these conditions HIF-α is stabilized, can form a heterodimer with HIF-1β in the nucleus and bind to the consensus cis-acting hypoxia response element (HRE) nucleotide sequence 5′-RCGTG-3′, which is present within the enhancers and/or promoters of HIF target genes (Fig. 1B) (33–35). HIF-α stabilisation therefore allows the cell to enact a transcriptional programme that is appropriate to the hypoxic environment (18) (Fig. 1B).

## 4. HIF target genes

The HIF heterodimer can regulate the expression of over 100 target genes involved in a broad range of physiological functions including: angiogenesis, erythropoiesis, metabolism, autophagy, apoptosis and other physiological responses to hypoxia (36). Canonical HIF signalling is based on the recognition of a putative HRE in the promoter or enhancer of the target gene that results in the recruitment of the HIF heterodimer and machinery required for transcription. Proteomics approaches have been used to identify protein changes in response to hypoxia in comparison with gene changes. Changes in just over 100 proteins in response to hypoxia have been identified (37,38). However, proteins identified represent both known and undescribed HIF targets, raising the possibility of HIF action outside of the conventional canonical pathway. Indeed, in addition to canonical signalling, there are various described mechanisms by which the stabilised HIF isoforms can influence the activity of other signalling pathways independent of the HIF heterodimer or a HRE. Non-canonical HIF signalling has been demonstrated to regulate aspects of Notch (39), c-Myc (40) and p53 (41) signalling.

## 5. Inflammation and the NF-κB pathway

Inflammation is a complex physiological process characterised by the activation of several coordinated signalling pathways in response to stress. Generally, the inflammatory response involves both anti- and pro-inflammatory mediators, given by the expression of small peptides (e.g., cytokines), glycoproteins (e.g., cluster of differentiation (CD)], and transcription factors, such as NF-κB.

**Figure 2:**
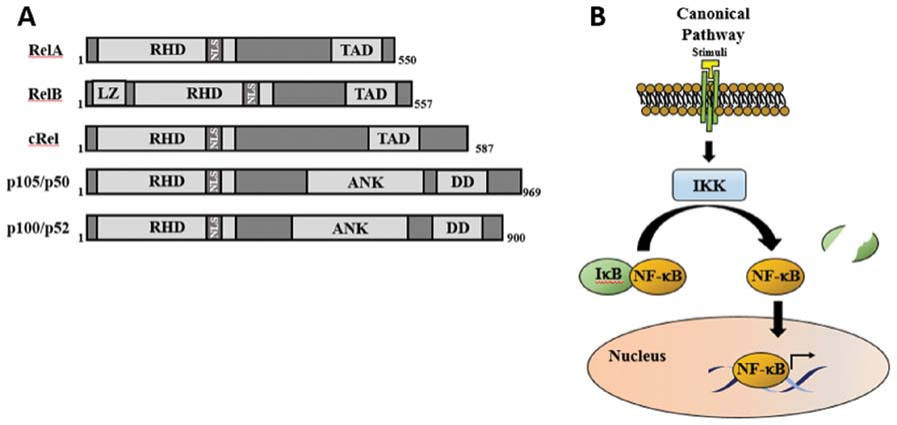
(A) Schematic diagram of NF-κB subunits. p50 and p52 are not shown, and they are derived from p105 and p100, respectively. Boxes represent different protein domains. (B) NF-κB canonical pathway. The presence of a stimuli results in the activation of the IKK complex, which mediates the phosphorylation of IκB protein, which signals it for proteasomal degradation. This results in NF-κB dimer release and translocation into the nucleus. NF-κB, nuclear factor-κB; IKK, inhibitor of κB kinase; RHD, Rel homology domain; TAD, C-terminal transactivation domain; LZ, leucine zipper motif; NLS, nuclear localisation signal; ANK, ankyrin-repeat motifs; DD, death domain.

NF-κB is considered the main pro-inflammatory family of transcription factors (42–44). In mammalians, it is characterised as a family of five Rel-domain proteins; RelA, RelB, cRel, p100/p52 and p105/p50 (Fig. 2A). Interestingly, it has been shown that almost all combinations of homo- or hetero-dimers between the five NF-κB subunits are possible (45). This is important, not only because it gives an extra layer of complexity to the NF-κB system, but also because it gives specificity according to cellular context, stimuli or DNA sequences that are bound to the subunits (44,46). All the NF-κB subunits are characterised by a conserved 300-amino acid domain, the Rel homology domain (RHD), which is located in the N-terminus of the protein (Fig. 2A), and is responsible for dimerisation, and DNA binding. While RelA, RelB and cRel contain a C-terminal transactivation domain (TAD) (Fig. 2A), p105 and p100 contain Ankyrin-repeats motifs in their C-terminus (ANK) (Fig. 2A), responsible for the dimerisation with other subunits, and subsequent sequestration/inactivation in the cytoplasm (Fig. 2B).

There are distinct pathways for the activation of NF-κB, according to the stimulus, as well as the kinases and NF-κB subunits involved (3). The most common, and most well studied is the classical or canonical NF-κB pathway (Fig. 2B). In unstimulated cells, the NF-κB dimers remain inactive in the cytosol, bound to an inhibitory protein, inhibitor of NF-κB (IκB) (47). Upon stimulation, for example by the pro-inflammatory cytokine, tumour necrosis factor-α (TNF-α), the inhibitor of κB kinase (IκB kinase; IKK), is activated, and phosphorylates IκB. This leads to the degradation of IκB and the release/translocation of the NF-κB complex into the nucleus (48). In the nucleus, the activated NF-κB complex binds to specific 9–10 base pair DNA sequences (κB sites) to activate a complex regulatory network in response to a specific stimulus (49). The combination of different possible homo- and heterodimers, stimuli and cellular context leads to a myriad of possible outcomes, namely the activation or inhibition of apoptosis, cellular growth and carcinogenesis (50).

The NF-κB system is complex and is involved in multiple biological roles; it is thus expected that it is deregulated in many different diseases. NF-κB abnormal activation has been associated with several human diseases, such as inflammation-related diseases (inflammatory bowel disease and asthma), cancer (apoptosis suppression), viral infections (HIV) and genetic diseases (incontinentia pigmenti) (51).

## 6. Crosstalk between hypoxia and inflammation in disease

Hypoxia and inflammation are intimately linked. It has been reported that individuals with mountain sickness presented with increased inflammatory cytokines circulating in the blood (52). Additionally, healthy volunteers who have been exposed to a hypoxic environment for three nights in high altitudes (>3,400 meters), presented with high levels of the inflammatory cytokine, interleukin (IL)-6, in the blood (53). On the other hand, several inflammatory diseases, such as RA and inflammatory bowel disease, also exhibit areas of combined hypoxia and inflammation, which are usually associated with a poor prognosis of the disease (54–57).

Hypoxia and inflammation are also connected at the molecular level (48,58,59). HIF (hypoxia) and NF-κB (inflammation) have been shown to have several common target genes, common regulators, and importantly, common stimuli (48). NF-κB activation has been shown to stabilise HIF-1α in hypoxia, and, together with HIF-1β, in inflammation (60,61). On the other hand, HIF-1α has been shown to repress NF-κB *in vivo* and *in vitro* under inflammatory conditions (59,62,63). The complexity of the combined response of HIF and NF-κB in hypoxia makes the crosstalk of these two pathways more intricate, and difficult to study. However, by developing a suitable inflammatory model, where the pathways can be controlled, as well as the conditions of the stimuli, these studies could provide very useful information that ultimately should be used to uncover new therapeutic strategies in a diverse range of diseases where hypoxia and inflammation are predominant features. In this review, the crosstalk between the main players induced in both inflammation and hypoxia in three clinical settings is addressed.

### Hypoxia and inflammation crosstalk in RA

**Figure 3:**
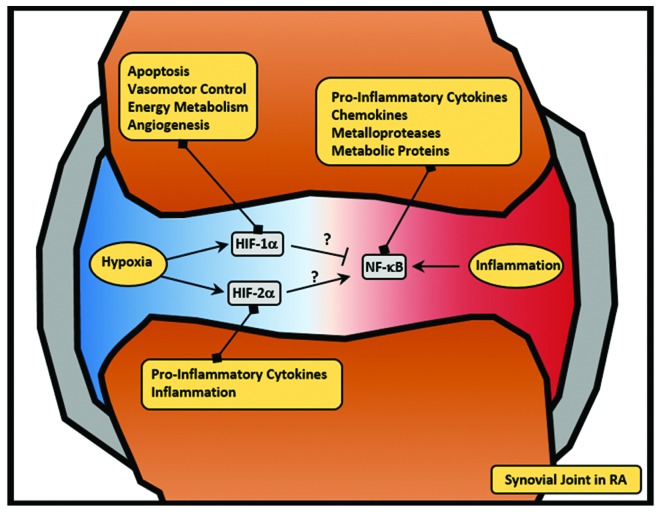
HIF and NF-κB crosstalk in RA. In RA, the synovial join is characterised by hypoxic and inflammatory regions (in blue and red, respectively). Hypoxia leads to the activation of HIF-1α, which is involved in several cellular processes (such as apoptosis, vasomotor control, energy metabolism and angiogenesis). Additionally, hypoxia leads to the activation of HIF-2α, which is involved in the activation of pro-inflammatory cytokines. In RA, inflammation leads to the activation of NF-κB, which activates a pro-inflammatory programme, including pro-inflammatory cytokines, chemokines, metalloproteases and metabolic proteins. While HIF-1α has been implicated in the repression of the NF-κB pathway (59), HIF-2α has been shown to increase inflammation (78). In RA, this crosstalk remains poorly understood. RA, rheumatoid arthritis; HIF, hypoxia-inducible factor; NF-κB, nuclear factor-κB.

RA is a systemic autoimmune disorder characterised by chronic inflammation of the synovial membranes of joint tissues at multiple anatomical sites which ultimately leads to localised destruction and debilitating deformity (64,65). The RA joint synovium is characterised by both inflammatory and hypoxic regions (Fig. 3), which are highly infiltrated with lymphocytes (CD4^+^ T cells, and B cells), macrophages and macrophage-like and fibroblast-like synoviocytes (66). The molecular basis of RA is still poorly understood, mainly because RA is a heterogeneous disease composed of several possible treatment responses, and clinical manifestations (67–69). These differences make RA difficult to treat, and further studies on the crosstalk between pathways involved in the disease are required.

### The role of NF-κB in RA

The deregulation of several transcription factors, such as NF-κB, activator protein-1 (AP-1), and signal transducer and activator of transcription (STATs), has been strongly associated with the inflammatory setting of RA (70–72). NF-κB, in particular, has been shown to be highly activated in the RA synovium (73,74). This is exceptionally important due to the major role of NF-κB in activating inflammatory responses, such as through the activation of the pro-inflammatory cytokine, TNF-α, or the chemokine, IL-8 (75). The activation of a coordinated and complex network of pro-inflammatory cytokines, chemokines, metalloproteases (MPPs) and metabolic proteins by NF-κB, leads to the activation of a positive feedback loop, enhancing the activation of more pro-inflammatory signals that ultimately results in chronic and persistent inflammation (Fig. 3) (75,76).

### The role of HIF in RA

The HIF family of proteins are additional transcription factors with direct relevance to RA (77,78). Recently, HIF-1α was identified as a key player in RA, and therefore as a potential therapeutic target (79). HIF is important to coordinate the hypoxia response in the synovial tissue, and the deregulation or failure of that response leads to cellular dysfunction, and can ultimately lead to cell death (80). Furthermore, the intense hypoxic region in the synovial tissue (2–4%), activates a hypoxic response through HIF, which is involved in regulating several genes involved in apoptosis, vasomotor control, energy metabolism, and importantly, angiogenesis (Fig. 3) (16,48,81–83).

Even though the role of HIF in RA has been firmly established, the contribution of each α-subunit remains poorly understood. Recently, HIF-2α was implicated as the essential catabolic regulator of inflammation in RA (78). In that study, the authors demonstrated that the overexpression of HIF-2α in joint tissues, but not HIF-1α, was sufficient to induce RA pathogenesis (78). The full contribution of the α-subunits to RA remains elusive. However, it seems clear that each α-subunit contributes differently to the progression of RA. HIF-1α plays a more anti-inflammatory role, whereas HIF-2α acts in a pro-inflammatory manner. What regulates this differential expression of the isoforms is still unknown. However, taking into consideration that NF-κB is the main activator of the HIF transcription factors, it would be interesting to understand whether NF-κB has any role in this HIF-1α to HIF-2α switch, and whether that would be dependent of the presence of hypoxia, inflammation, or both combined.

### Inflammatory bowel disease (IBD)

**Figure 4:**
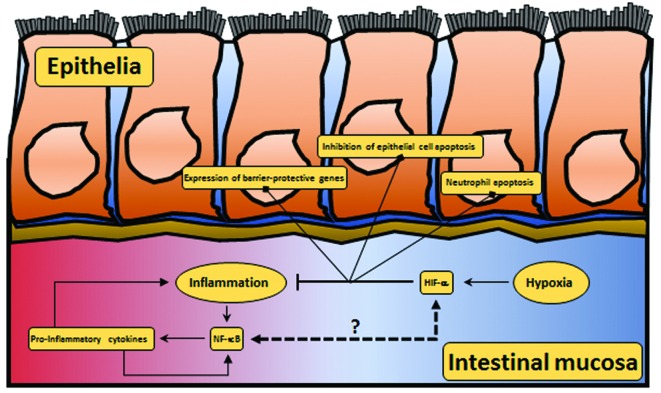
HIF and NF-κB crosstalk in inflammatory bowel disease. In IBD, the intestinal mucosa is characterised by hypoxic and inflammatory regions (in blue and red, respectively). HIF-1α is activated in hypoxia, and acts as a protective barrier by inhibiting apoptosis of epithelial cells, enhancing the barrier-protective genes, and by promoting the apoptosis in neutrophils. Inflammation leads to the activation of NF-κB, which is involved in the expression of inflammatory cytokines that can lead to inflammation and/or NF-κB activation. HIF, hypoxia-inducible factor; NF-κB, nuclear factor-κB; IBD, inflammatory bowel disease.

The intestinal mucosa is exposed to steep hypoxic gradients (63) and is in a constant state of controlled inflammation, which is necessary to allow tolerance to otherwise harmless ingested dietary antigens (Fig. 4) (84). This fine balance is pathologically disturbed in inflammatory bowel disease (IBD); a relapsing-remitting progressive disorder of the gastrointestinal tract that comprises both Crohn’s and ulcerative colitis. The symptoms of IBD can range from mild to severe and include abdominal pain, intestinal bleeding, weight loss, fever and diarrhoea (85). The two IBD sub-types have different distribution patterns: ulcerative colitis is restricted to the colon, whereas Crohn’s colitis can affect any part of the GI tract. Both are thought to occur when inappropriate immunological activity in the intestinal mucosa results in epithelial barrier dysfunction leading to exposure of the mucosal immune system to luminal antigenic material and further cycles of inflammation and barrier dysfunction that underlie disease progression (86,87).

### Role of the HIF system in IBD

Hypoxia has been found to play a role in IBD. Lower resting oxygen levels have been demonstrated in sections of IBD tissue compared to the controls using a 2-nitroimidazole based approach (63). In keeping with these observations, HIF-1α and HIF-2α activation has been associated with disease and increased vascular density in human specimens (88). The increased vascular density was subsequently demonstrated to be effected by vascular endothelial growth factor (VEGF), an established target of the HIF system (89). Compartmental analyses of the effects of hypoxia have been possible in murine models, where hypoxia has been shown to affect the epithelium, primarily during periods of inflammation (63). The colonic epithelium is the most hypoxic and HIF-active tissue layer because it is physically farthest away from the colonic vascular plexus and closest to the anoxic bowel lumen. This effect is exacerbated by oxygen consumption by luminal bacteria (90), and the presence of inflammatory mediators and lipopolysaccharide (LPS), which have been shown to regulate HIF activity (48).

In the context of IBD, HIF system activity is thought to be protective, acting through three mechanisms: i) inhibition of epithelial cell apoptosis; ii) enhanced expression of barrier-protective genes; and the iii) promotion of neutrophil apoptosis (Fig. 4) (86). Evidence of the anti-apoptotic effects of HIF has been demonstrated indirectly through experiments to investigate the role of the hydroxylase inhibitor, dimethyloxaloylglycine (DMOG), in colitis. Using a murine model of dextran sodium sulfate (DSS)-induced colitis, HIF stabilisation following treatment with DMOG has been shown to prevent apoptosis in a mechanism thought to be mediated by the anti-apoptotic protein, cIAP-2 (91). Recently, this effect has been specifically attributed to PHD1, since the homozygous loss of PHD1, but not PHD2 or PHD3, has been shown to be protective in the same murine model of DSS-induced colitis (92). This effect is most likely HIF-dependent, since the conditional knockout of HIF-1α in mouse intestinal epithelial cells has been shown to result in an enhanced susceptibility to the development of colitis (63).

In addition to its anti-apoptotic effects, the HIF system can protect against colitis through the expression of barrier-protective genes. Several HIF-dependent target genes have been proposed as mediators of this effect: CD55 (93), ecto-50 nucleotidase (94), A2B receptor (95) MUC-3 (96), intestinal trefoil factor (97), and P-glycoprotein (98) all play a role in the regulation of the intestinal mucosa barrier and have all been demonstrated to be regulated in a hypoxia-dependent manner.

There is also evidence of the differential effects of the HIF-α isoform in IBD. HIF-2α expression has been shown to be increased in colon tissues of mice after the induction of colitis. This was also observed in patients with ulcerative colitis or Crohn’s disease (62). Interestingly, in that study, while the loss of HIF-2α was associated with attenuated colonic inflammation, the overexpression of HIF-2α led to spontaneous colitis and increased inflammation.

### Role of NF-κB in IBD

Other transcriptional programs are active in IBD in addition to those enacted by the HIF system. IBD is primarily an inflammatory pathology and NF-κB activity has been linked to its progression (5). A high degree of NF-κB induction has been demonstrated in intestinal macrophages and epithelial cells (99). In IBD, inflammatory cytokines can drive NF-κB activation, leading to the production of more inflammatory cytokines and potentiating further NF-κB activation (Fig. 4). NF-κB-induced TNF-α expression is one example of this type of positive feedback loop (100). Interestingly, NF-κB can have a dual role in IBD, potentiating inflammation in intestinal macrophages while protecting from inflammation in mucosal epithelial cells. Sharing interesting similarities to the effects of HIF activation, NF-κB signalling in intestinal epithelial cells has been shown to be protective against the development of colitis (101). Deletion of the NF-κB pathway in intestinal epithelial cells results in decreased expression of anti-apoptotic genes, such as Bcl-xL, and leads to reduced epithelial barrier function and increased susceptibility to colitis (102). Conditional knockout of NEMO and subsequent NF-κB inhibition has been shown to result in severe epithelial inflammation in a murine model (102). Similarly, epithelial cell-specific IKKβ deletion has been shown to result in the sustained production of pro-inflammatory Th1 cytokines and increased intestinal inflammation (103). Several treatments have been proposed to target NF-κB activity in IBD, including proteasome blockade, the administration of non-coding RNAs to interfere with NF-κB-DNA binding and anti-TNF-α immunotherapy. However, all have been met with significant systemic toxicity due to the broad role of NF-κB in multiple organs.

### NF-κB-HIF crosstalk in IBD

Sharing similarities with the microenvironment of RA, in IBD both inflammation and hypoxia are present in the intestinal epithelium and contribute to disease progression (104). It is generally [but not universally (105)] understood that both NF-κB and HIF activity are protective in episodes of colitis (101). Significant crosstalk between these pathways has already been established, and it has been proposed that both pathways may act in concert to contribute to the epithelial barrier function of the colon in a process that is deregulated in IBD.

One example of this crosstalk is the regulation of apoptosis by both pathways (106,107). The caspase recruitment domain family, member 9 (CARD9) is understood to function as a molecular scaffold for the assembly of a BCL10 signalling complex that activates NF-κB (106), and has also been shown to be involved in the regulation of hypoxia-sensitive pathways (107). CARD9 therefore represents one point of crosstalk that may be important in the development of IBD as a promising target for further investigation.

Our laboratory and others have demonstrated NF-κB-dependent HIF-1α mRNA regulation (61,108). NF-κB can also regulate HIF signalling through IKKγ and HIF-2α, which increases HIF-2α transcriptional activity through interaction with cAMP response element-binding (CREB) binding protein (CBP)/p300 (109). Negative feedback through the NF-κB-dependent induction of the micro-RNA, *miR-155*, in response to LPS has been shown to target HIF-1α for silencing (110). Furthermore, our laboratory have recently demonstrated an evolutionarily conserved negative feedback mechanism through which HIF can regulate NF-κB in a mechanism that is dependent on the kinases, TAK-IKK and CDK6 (59).

### CRC

CRC is a lethal disease affecting over 500,000 individuals annually (111). In contrast to the protective effects of HIF and NF-κB activity in IBD, both can play important roles in the development of colorectal malignancy. In CRC, the hypoxic milieu is similar to that of IBD but, critically, the cells are transformed to allow them to react differently to the activation of either system. In addition, chronic inflammation is a hallmark of cancer (112). The role of the HIF system and the role of NF-κB activity are considered below, and the significance of their crosstalk with respect to the development of CRC is examined.

### Role of the HIF system in CRC

**Figure 5:**
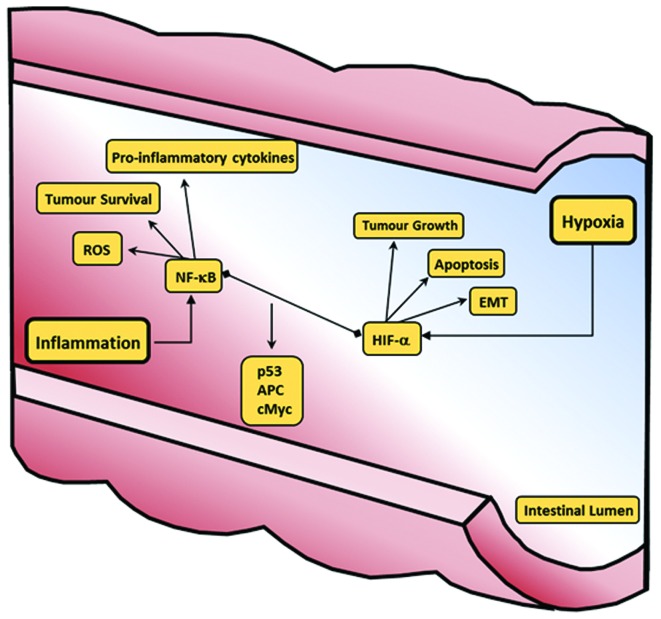
HIF and NF-κB crosstalk in CRC. In CRC, the intestinal lumen is characterised by hypoxic and inflammatory regions (in blue and red, respectively). HIF-1α is activated in hypoxia, and is involved in the modulation of tumour growth, apoptosis, and EMT. Inflammation leads to the activation of NF-κB, which is involved in the expression of pro-inflammatory cytokines, ROS production, and tumour survival. In CRC, there are some points of crosstalk between NF-κB and HIF, namely in the regulation of p53, APC, and cMyc. HIF, hypoxia-inducible factor; NF-κB, nuclear factor-κB; CRC, colorectal cancer; EMT, epithelial to mesenchymal transition; ROS, reactive oxygen species; APC, adenomatous polyposis coli.

The role of the HIFs in cancer progression has long been appreciated due to their ability to promote angiogenesis through one of the principally identified HIF-1α target genes, VEGFA (113). However, it is becoming more evident that hypoxia and the HIF system can affect tumour growth through modulation of proliferation, apoptosis and epithelial to mesenchymal transition (EMT) (Fig. 5). Hypoxia and the subsequent HIF activation are generally understood to be prognostically bad and lead to tumour progression (114). In CRC, HIF-1α stabilisation has been shown to lead to a poor disease outcome. Shay *et al* (114) demonstrated that the inhibition of HIF signalling using acriflavine halted the progression of an autochthonous model of established colitis-associated colon cancer in immuno-competent mice. In their model, treatment with acriflavine was shown to decrease tumour number, size and advancement, in an effect thought to be mediated through the inhibition of HIF-dependent targets, such as VEGFA. These data provide a direct link between HIF-1α expression and tumour progression. However, HIF isoform activation can be antagonistic in the context of tumour progression. In contrast to the effect of high HIF-1α expression, high HIF-2α expression has recently been reported to prevent CRC progression (115). The antagonistic effects of HIF-1α and HIF-2α are important for the regulation of proliferation and apoptosis in cancer biology (40,82,116). The HIF system can affect proliferation through the regulation of cMyc. HIF-1α can promote cell cycle arrest by the direct opposition of c-Myc activity and the induction of p21 in CRC (116). Conversely, HIF-2α has been shown to promote proliferation in through its augmentation of cMyc function (40).

The HIF system can also affect apoptosis through the regulation of p53. p53 stability leads to apoptosis in somatic cells and it is frequently mutated in cancers in pursuit of immortality. HIF-1α has been shown to stabilise wild-type p53 via physical interaction through its ODD (41,117). As a form of negative feedback, p53 can promote the degradation of HIF-1α (118). The negative feedback of wild-type p53 on HIF-1α could explain the increased stability of HIF-1α in tumours that express mutant p53 which is incapable of degrading HIF-1α. The net result of the p53-HIF-1α interaction is increased apoptosis in damaged cells that are exposed to hypoxia (119). HIF-2α can inhibit p53 phosphorylation, resulting in a reduction in p53 pathway activity and the prevention of apoptosis in response to damaging stimuli (120). In addition to its role in the regulation of p53, HIF has been linked to the positive regulation of apoptosis through the control of several pro-apoptotic factors, including caspase-3, Fas and Fas ligand (121).

Hypoxia is a critical determinant of the motile and invasive phenotype of cancer cells. HIF activation is also important in the regulation of genes involved in EMT, including the direct regulation of the EMT-promoting transcription factors, Snail and Twist, which have both been described as direct targets of the HIF system (122–124). EMT is a critical event in the induction of tumour metastasis (125). Notch has also been shown to mediate HIF-1α-dependent EMT (126).

### Role of NF-κB in CRC

The role of NF-κB in CRC is an active area of study (101,127–129). Inflammation is an important trigger in the establishment and development of CRC. Patients with long-standing IBD have an increased risk of developing CRC (127,128). In this context, NF-κB activation can promote tumourigenesis and CRC progression. In CRC, chronic inflammation results in sustained reactive oxygen species (ROS) production, leading to DNA damage (Fig. 5) (130). Treatment with non-steroidal anti-inflammatory drugs (NSAIDs) reduces the development of CRC in patients with IBD and hereditary CRC (131,132), and the inactivation of NF-κB signalling reduces the formation of inflammation-associated tumours (101,129). IL-6 has been shown to be important for the number and size of tumours formed in mice (133), and IKKβ conditional knockout mice have been shown to develop more numerous tumours (134).

As with the HIF system, the mechanism of NF-κB-induced tumourigenesis and progression can be multifactorial. The activation of the NF-κB pathway confers survival, proliferation, angiogenic and migratory advantages (Fig. 5) (112,135–138); all of which are hallmarks of cancer (112). NF-κB activation can block apoptosis by regulating the anti-apoptosis proteins, such as inhibitor of apoptotic proteins (IAPs) (139), or by the inhibition of prolonged c-Jun N-terminal kinase (JNK) signalling, modulating the accumulation of ROS (140). Alternatively, NF-κB activation can enhance IL-2 production, which can activate Janus kinase 3 (Jak3) by autophosphorylation (141). Jak3 can activate STAT3. Jak3 and STAT3 over-activation has been observed in human colon cancer *in vivo* and *in vitro*, and shown to prevent apoptosis, leading to poor prognosis (142,143). In addition, NF-κB activation can affect proliferation and cell growth through the regulation of its target genes, cyclin D1 and cMyc (144–146), and promote angiogenesis through the regulation of VEGF and IL-8 (136). Finally, NF-κB activation has been shown to affect the expression of matrix metalloprotease-9 (MMP-9), in murine colon adenocarcinoma cells (147), an important protein in the regulation of migration and invasion.

### NF-κB-HIF crosstalk in CRC

The data presented above demonstrate a clear overlap between the effectors of the HIF and NF-κB systems in the establishment and development of CRC. Solid tumours are characterised by the presence of hypoxia, as well as inflammation (6). Potential points for crosstalk include the regulation of cMyc and p53 (Fig. 5). NF-κB interacts with the co-activators, p300 and CREB-binding protein, to inhibit p53 function. This effect is reinforced by the NF-κB-dependent upregulation of the p53 inhibitor, mouse double minute 2 (MDM2) (3,148) and is similar to that exerted on p53 by HIF-1α. The expression of NF-κB, HIF, VEGF and Bcl-3 has been shown to correlate with proliferation, angiogenesis, decreased survival and a poor clinical outcome (149,150). In addition, TNF-α has been shown to stabilise Snail and β-catenin in a process that requires the downregulation of glycogen synthase kinase-3β (GSK3β) by NF-κB and the activation of Akt cascades, resulting in the promotion of EMT (151). These data are clinically important since NF-κB and Twist have been associated with lymph node metastasis in patients with CRC (152). Interestingly, HIF has been shown to interact with both Snail and Twist, making this another potential point for crosstalk between the pathways (153).

In addition to the mechanisms outlined above, there is a complex interplay between HIF, NF-κB and adenomatous polyposis coli (APC), that appears to be important in CRC (Fig. 5). One of the earliest events in the development of CRC is loss of the APC gene. Our laboratory has recently reported a functional crosstalk between HIF-1α and APC at the transcriptional level (154). HIF activation represses APC expression, acting at its promoter to result in positive activation and proliferation through the Wnt/β-catenin signalling and the TCF-LEF pathway (155), reduction in genetic and microtubule stability and reductions in cell migration (6,156).

The repression of APC by HIF-1α is complicated by the fact that medium levels of β-catenin can induce NF-κB, resulting in positive feedback, and high levels of β-catenin inhibit NF-κB, resulting in negative feedback (6). Further studies are required to determine the functional significance of this interaction *in vivo*. However, it represents another exciting point of crosstalk with importance for CRC disease progression.

## 7. Conclusion

In this review, the current understanding of the mechanisms of the HIF and NF-κB systems has been discussed with specific reference to the crosstalk between these two stress-responsive pathways. This crosstalk is significant for many disease processes and its role in RA, inflammatory bowel disease and CRC has been discussed in detail. It is important to note that the crosstalk between these pathways has significance beyond pathological processes. For example, in healthy individuals who live at a high altitude, prolonged HIF activation can lead to reduced NF-κB activity, effectively dampening the immune response. Further studies in this area is required; however, it is interesting that the anecdotal evidence of increased *H. pylori* infection in Tibetan monks exists (157). Individuals with mountain sickness have presented with increased levels of inflammatory cytokines circulating in the blood (52). Another study demonstrated that healthy volunteers who spent three nights at high altitudes (>3,400 meters), presented with high levels of the inflammatory cytokine, IL-6 (53). This hypoxia-inflammation crosstalk is also relevant in the clinical context. It was shown that ischemia in organ grafts increased the risk of inflammation and, consequently, graft failure or organ rejection (158). Accurate systematic experimentation is important to determine the mechanisms of the crosstalk between these pathways since these findings may have an impact on multiple disease processes, apart from those discussed herein. These include diabetes and systemic sclerosis, where limb perfusion is not optimal, resulting in increased tissue breakdown in the absence of an appropriate inflammatory response, leading to an increased infection rate.

## Abbreviations

IKK: inhibitor of κB kinase
NF-κB: nuclear factor-κB
HIF: hypoxia-inducible factor
ARNT: aryl hydrocarbon nuclear translocator
PHD: prolyl hydroxylase
vHL: von Hippel Lindau
TNF: tumour necrosis factor
